# A cellular senescence-related genes model allows for prognosis and treatment stratification of cervical cancer: a bioinformatics analysis and external verification

**DOI:** 10.18632/aging.204981

**Published:** 2023-09-27

**Authors:** Weiwei Yang, Lijuan An, Yanfei Li, Sumin Qian

**Affiliations:** 1 Gynecology Department 2, Cangzhou Central Hospital, Yunhe District, Cangzhou 061000, Hebei Province, China

**Keywords:** cervical cancer, senescence, TCGA, prognostic model, nomogram

## Abstract

Background: Cervical cancer (CC) is highly lethal and aggressive with an increasing trend of mortality for females. Molecular characterization-based methods hold great promise for improving the diagnostic accuracy and for predicting treatment response.

Methods: The mRNAs expression data of CC patients and cellular senescence-related genes were obtained from the Cancer Genome Atlas (TCGA) and CellAge databases, respectively. Differentially expressed genes (DEGs) of senescence related genes between tumor and normal tissues were used for Least absolute shrinkage and selection operator (LASSO) regression to construct a prognostic model. Univariate and LASSO regression analyses were applied to establish a predictive nomogram. The performance of the nomogram were evaluated by Kaplan-Meier curve, receiver operating characteristic (ROC), Harrell’s concordance index (C-index), and calibration curve. GSE44001 and GSE52903 were used for external validation.

Results: We established a cellular senescence-related genes-based stratified model, and a multivariable-based nomogram, which could accurately predict the prognosis of CC patients in the TCGA database. The Kaplan–Meier curve indicated that patients in the low-risk group had considerably better overall survival (OS, *P* =2.021e-05). The area under the ROC curve (AUC) of this model was 0.743 for OS. Multivariate analysis found that the 6-gene risk signature (HR=3.166, 95%CI: 1.660-6.041, *P*<0.001) was an independent risk factor for CC patients. We then designed an OS-associated nomogram that included the risk signature and clinicopathological factors. The AUC reached 0.860 for predicting 5-year OS. The nomogram showed excellent consistency between the predictions and actual survival observations. Two external GEO validations were corresponding to the gene expression pattern in TCGA.

Conclusions: Our results suggested a six-senescence related signature and established a prognostic nomogram that reliably predicted the overall survival for CC. These findings may be beneficial to personalized treatment and medical decision-making.

## INTRODUCTION

Cervical cancer (CC) is the fourth leading cause of cancer death in women and was diagnosed in approximately 570,000 patients worldwide in 2018, resulting in 31,000 deaths [1]. Early-stage CC can be treated by traditional chemotherapy, radiation therapy, or surgery, but late-stage CC patients often develop resistance to radiotherapy and their prognosis varies depending on the stage of the cancer [2]. Therefore, it is necessary to improve the survival rate of CC patients by exploring new prognostic markers and therapy strategies.

Cellular senescence refers to a state of permanent and irreversible growth arrest in cells, which occurs in response to various internal and external factors such as telomere dysfunction and oncogene activation [3, 4]. This process is closely associated with tumorigenesis and development [5]. Recent studies have highlighted a subtle relationship between cellular senescence and the aging phenotype, characterized by an irreversible growth arrest, morphological changes, and the production of senescence-associated secretory phenotype (SASP) [6]. In the past, senescence has been recognized as an adaptive response of cells to adverse conditions. In the context of cancer, senescence-mediated growth stagnation serves as an intrinsic mechanism to counteract tumor progression by preventing the proliferation of (pre)neoplastic cells [7]. Pereira et al. and other researchers have shown that senescent cells can avoid immune clearance by discharging SASP factors, such as IL-6. This, in turn, leads to the upregulation of HLA-E, hindering the removal of natural killer (NK) cells and T cells within premalignant lesions [8]. Given the importance of cellular senescence in tumors, a multitude of studies have investigated the expression of SRGs in cancer and devised prognostic models to predict survival outcomes. Nonetheless, the prognostic significance of senescence and its immune-mediated functions within the context of CC remains inadequately comprehended.

The development of biotechnology has made new methods such as bioinformatics analysis widely available. This has resulted in important contributions to tumor diagnosis and prognosis prediction by identifying candidate biomarkers and exploring the molecular mechanisms of cancer. RNA sequencing can be used to identify splice variants, unmapped genes, and spliced unidentified noncoding RNAs. Using the dataset of The Cancer Genome Atlas (TCGA) for CC, a risk prediction model was built based on 3 autophagy-related genes (CHMP4C, FOXO1, and RRAGB), and can be used to effectively predict the prognosis of CC patients [9]. New biomarkers for prognosis prediction of CC are required to develop advanced therapy strategies and further improve therapeutic treatments. In this research, we gathered the expression profiles and clinical data of patients diagnosed with CC. From the aging database, we identified several senescence-related genes (SRGs) and selected differentially expressed genes (DEGs) associated with senescence for further analysis. Using LASSO regression, we developed a prognostic multigene signature based on these DEGs in the TCGA cohort. We then validated the signature in an independent GEO cohort. Additionally, we constructed a predictive nomogram to assist in predicting the prognosis of CC patients, which was further validated using data from the TCGA cohort.

## MATERIALS AND METHODS

### Acquisition and procession of TCGA and senescence-related gene set

From the TCGA website (https://portal.gdc.cancer.gov/), we acquired gene expression data, transcriptome profiles, and clinical information of CC patients. We obtained a curated gene list consisting of 279 cellular senescence-related genes from the CellAge database (https://genomics.senescence.info/cells/, seen in Supplementary Table 1). Using the criteria of log_2_(FC) >1 and *P*-value < 0.05, we performed differential expression analysis of these differentially expressed senescence-related genes (DE-SRGs) in three hundred and six CC and three normal tissues using the “edgeR” package in R 3.6.1 software. For validation, we obtained two datasets, GSE44001 and GSE52903, from the GEO database. We visualized the expression levels of the prognosis-associated DE-SRGs by employing the “pheatmap” and “ggplot” packages in R, respectively, while comparing normal and tumor samples. Our study adhered to the publication guidelines provided by TCGA.

### Senescence-related gene analysis using consensus clustering

Using the “ConsensusClusterPlus” package in R, we performed an unsupervised clustering analysis of SRGs. First, the cumulative distribution function (CDF) curve showed a gradual and smooth increase. Second, each subgroup had an appropriate sample size. Finally, the correlation within each subgroup increased while the correlation between subgroups decreased. Additionally, we performed PCA using the “prcomp” command in R to further explore the data structure and patterns.

### Development and validation of the prognostic signature related to senescence

Next, we employed LASSO regression analysis using the “glmnet” R package to identify key prognosis-related DE-SRGs. The risk score formula for predicting the prognosis of CC patients is as follows:


$$\text{Risk}\;\text{score}=\sum {\text{n}}_{\text{i}}=\sum \left(\text{Coefi}*{\text{x}}_{\text{i}}\right)$$

The regression coefficient of DE-SRGs in the LASSO analysis was denoted as *Coefi*. *Xi* represents the expression value of the DE-SRGs, and *n* represents the number of prognostic DE-SRGs. Patients were classified into low- and high-risk groups based on the median value of the risk score. Kaplan–Meier survival analysis of these groups was conducted using the “survival” package in R. For evaluating the sensitivity and specificity of the risk signature, we utilized the “timeROC” package in R software to construct the ROC curve.

### Development and validation for nomogram including risk score model

We conducted univariate and multivariate Cox regression analyses to ascertain whether the risk signature served as an independent risk factor for CC patients. Subsequently, a nomogram comprising these independent prognostic factors was constructed using the “rms” package. Calibration curves were generated, and the C-index was measured to validate the predictive capability of this signature. We further assessed the prognostic performance of the nomogram using Kaplan-Meier survival analysis and the area under the time-dependent ROC curve (AUC).

### Bioinformatics analysis of SRGs risk model

In order to reveal the function of risk signature, we utilized GSEA (http://software.broadinstitute.org/gsea/index.jsp) [10] to assess the significant differences in identified gene sets between the low and high groups [11]. For GSEA analysis, we utilized the hallmarker.all.v6.2.symbols.gmt collection of annotated gene sets from the Molecular Signatures Database as our reference gene sets in GSEA software, with a significance cutoff set at P<0.05. Additionally, to investigate the potential biological functions of differentially expressed SRGs, we conducted Gene Ontology (GO) and Kyoto Encyclopedia of Genes and Genomes (KEGG) pathway enrichment analysis using the “clusterProfiler” package in R [12]. Functional categories with an adjusted P-value of less than 0.05 were regarded as significant pathways.

### External validation of the DE-SRGs

To validate the expression patterns of the selected genes in the TCGA dataset, we extracted mRNA expression data from GEO datasets (GSE44001 and GSE52903) for further analysis. The differential expression of these selected genes between CC and non-tumor tissues was assessed using Prism 7.0 (GraphPad, San Diego, CA, USA) through the Wilcoxon signed-rank test, with a significance level set at *P* < 0.05. Additionally, we generated survival and ROC curves to evaluate the performance of the risk signature.

### Statistical analysis

R was utilized for all data analyses. The AUC was employed as a metric to evaluate the prognostic accuracy. The correlation between risk scores and the estimation of stromal and immune cells in ESTIMATE score was assessed using Pearson correlation. Statistical significance was defined as a *P*-value of <0.05 in all analyses.

### Availability of data and materials

The datasets analyzed during the current study are available in the TCGA repository, (https://portal.gdc.cancer.gov/. Accession No. TCGA-CC).

## RESULTS

### Acquisition of senescence-related DEGs in CC patients

**Figure 1:**
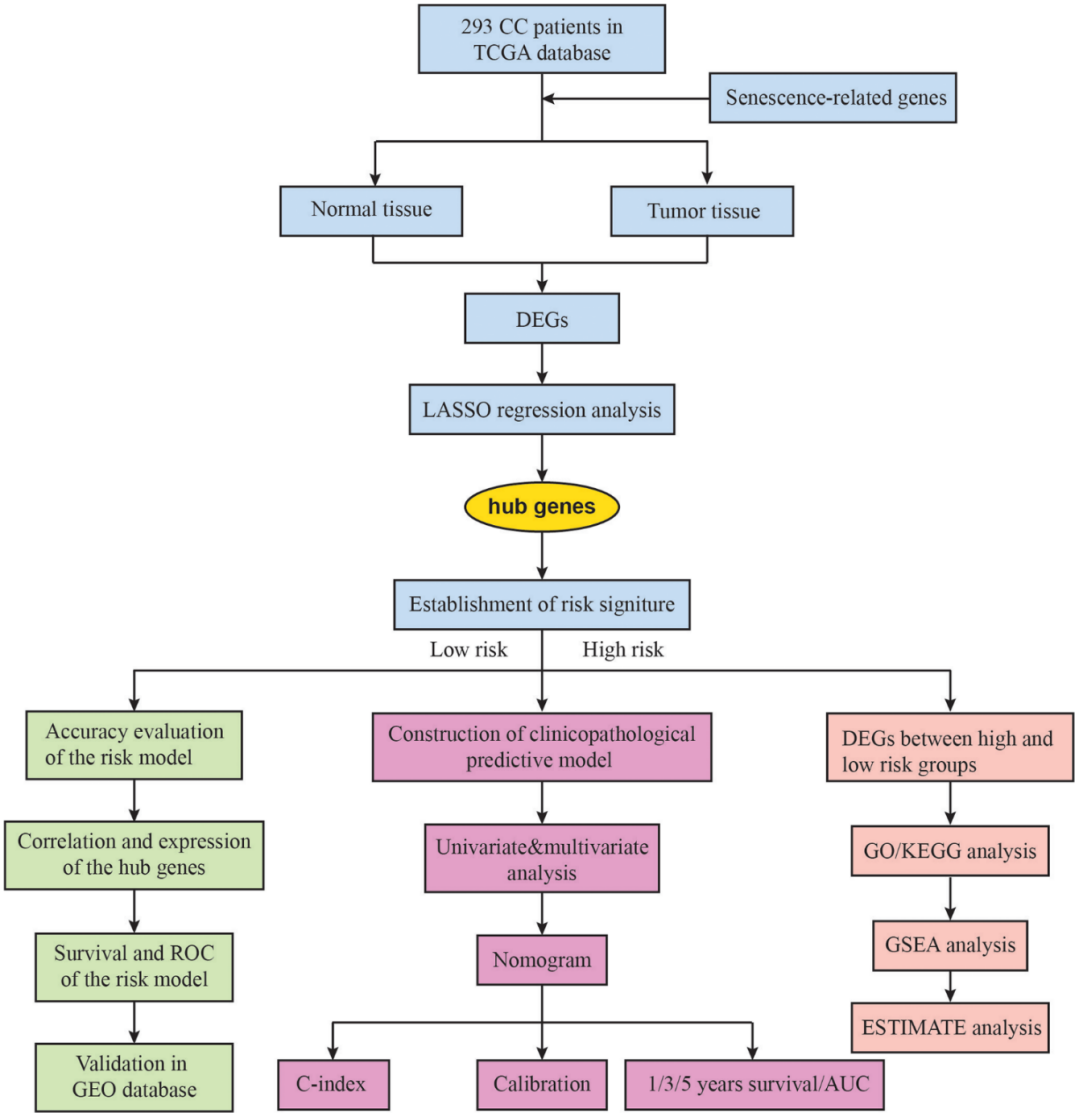
**The flow chart of the study design.** CC, cervical cancer; TCGA, The Cancer Genome Atlas; DEGs, differentially expressed genes; LASSO, Least Absolute Shrinkage and Selection Operator; AUC, area under the ROC curve; GO, gene oncology; KEGG, Kyoto Encyclopedia of Genes and Genomes; GSEA, gene set enrichment analysis.

**Figure 2:**
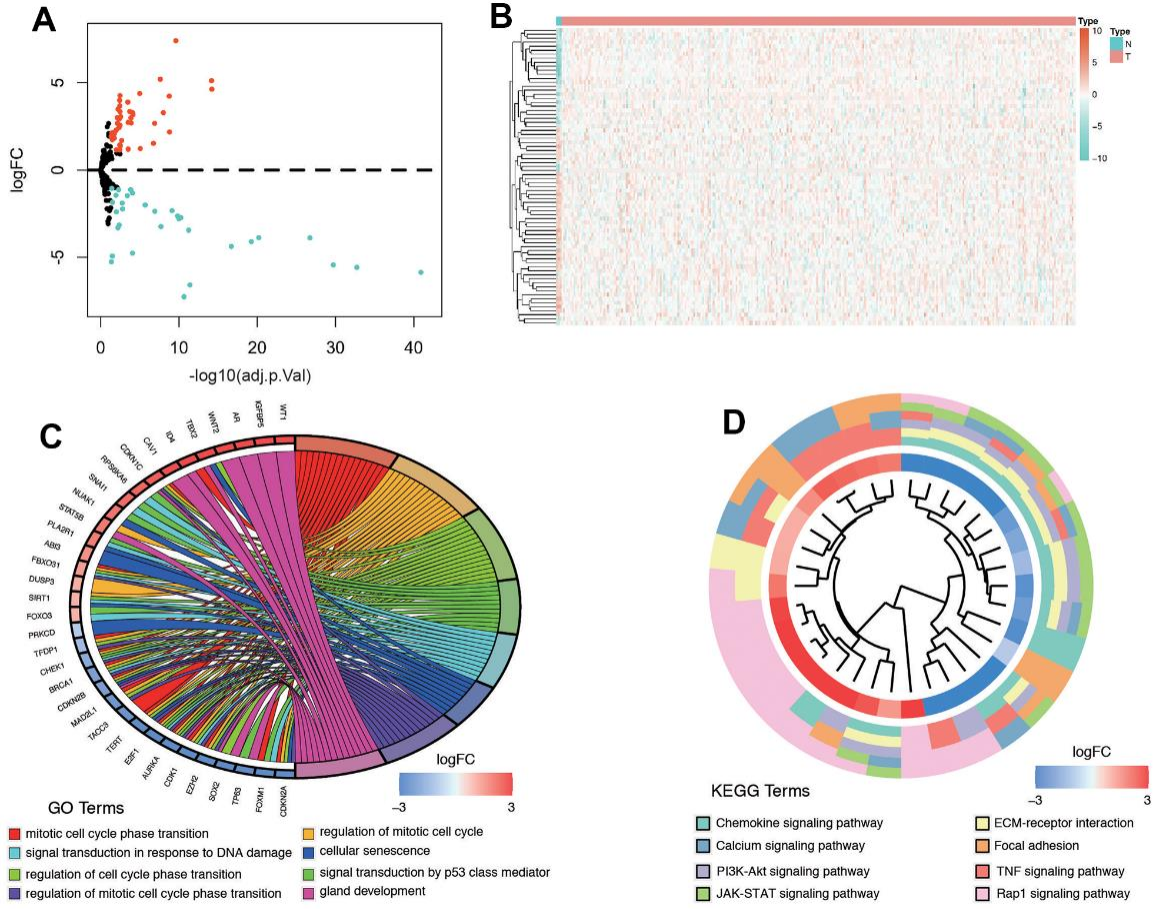
**Differentially expressed senescence-related genes and functional analysis in cervical cancer.** (**A**) Volcano plot. (**B**) Heatmap. (**C**) GO analysis of DEGs. (**D**) KEGG analysis.

Figure 1 illustrates the comprehensive flow chart detailing the construction of the predictive model in this study. From the senescence-related genes in the database, we extracted a total of 279 senescence-related genes. After the integration of the SRGs expression data of 306 TCGA tumor tissues and 3 normal tissues, we obtained 42 upregulated and 32 downregulated SRGs (Figure 2A, 2B). The 74 DE-SRGs are shown in Supplementary Table 2. GO and KEGG analysis of the DEGs are shown in Figure 2C, 2D.

### Identification of senescence subtypes in cervical cancer

**Figure 3:**
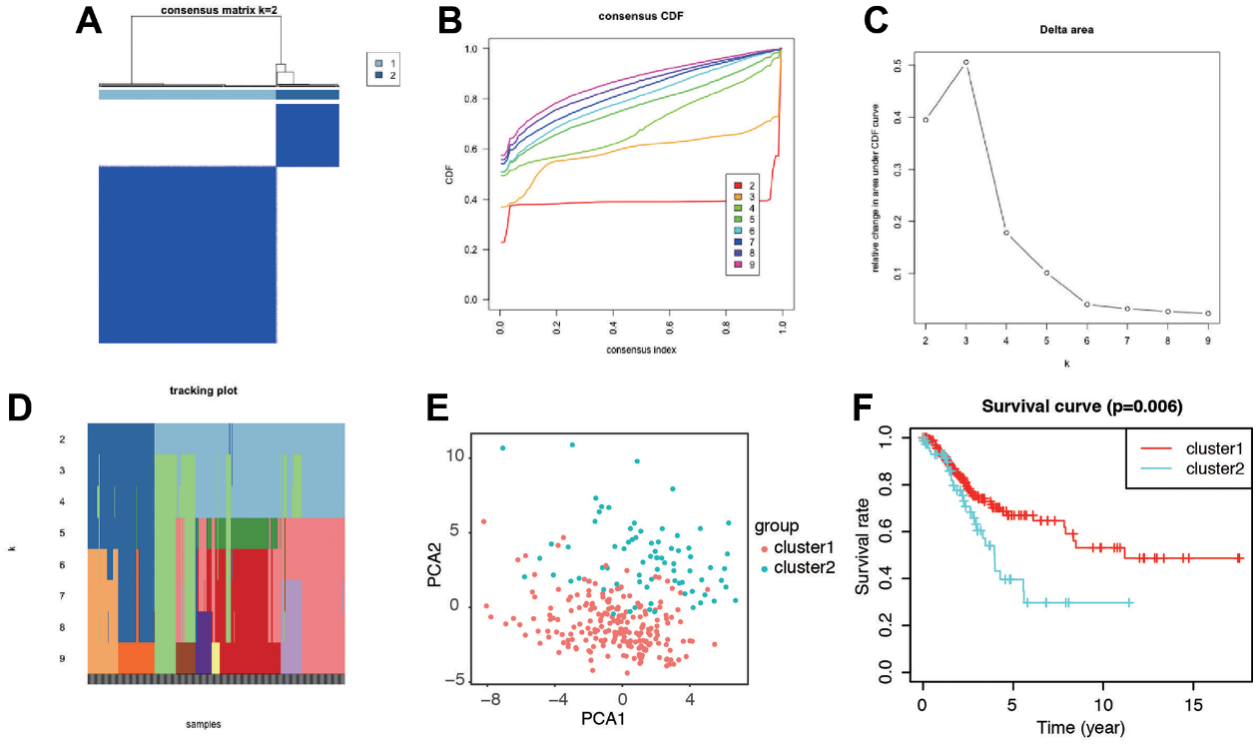
**Identification of the molecular subtypes of the CC patients using the DEGs associated with senescence.** (**A**) The CC patients were stratified into 2 clusters based on the consensus clustering matrix (k=2). (**B**–**D**) Consensus clustering model with cumulative distribution function (CDF) by k from 2 to 9. (**E**) The results of PCA analysis among the two subtypes. (**F**) Survival curves of patients in the two clusters.

To gain further insights into the characteristics of senescence-related genes in cervical cancer, we employed a consensus clustering algorithm to classify patients based on the expression profiles of 279 senescence-related genes. Through this analysis, we identified two distinct clusters, with k=2 being the optimal choice (Figure 3A–3D). PCA analysis revealed clear separations in the distribution of senescence-related genes between the two clusters (Figure 3E). Importantly, patients in cluster 1 exhibited a significantly longer OS compared to those in cluster 2, as demonstrated by Kaplan–Meier analysis (log-rank test, *P* = 0.006; Figure 3F). These findings motivated us to delve deeper into the association between senescence and prognosis in CC patients by investigating the expression patterns of senescence-associated genes.

### Construction of prognostic markers of cervical cancer

**Figure 4:**
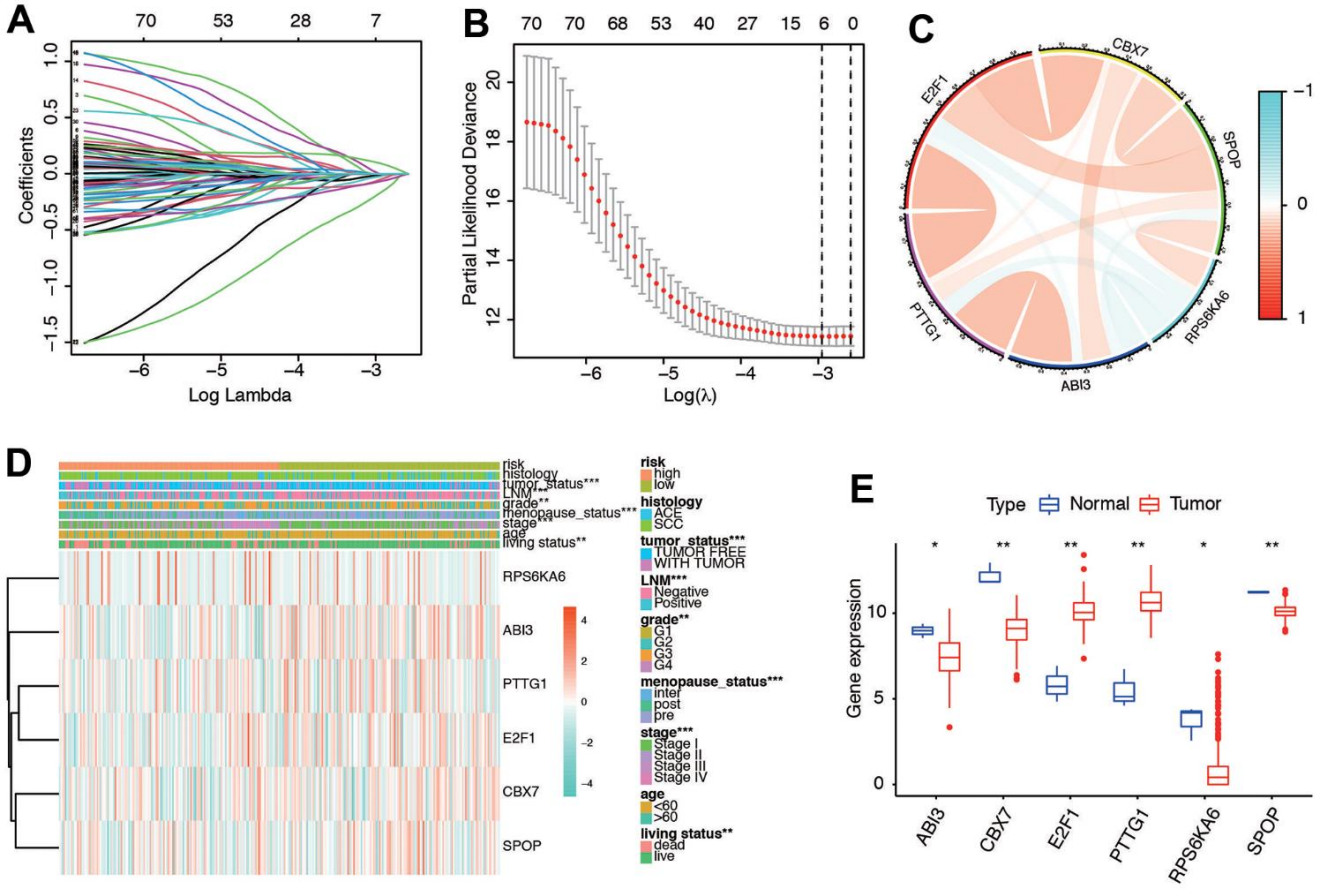
**Ten-fold cross-validation for tuning parameter selection and a gene expression.** (**A**) Plots of the ten-fold cross-validation error rates. (**B**) LASSO coefficient profiles of the six senescence-related genes. (**C**) Relationships between the six genes. (**D**) Gene expression of the six genes and clinicopathological characteristics in different risk groups. (**E**) The expression of six genes in normal and tumor tissues.

We merged the expression profiles of SRGs and clinical follow-up information of CC to identify a total of CC samples. Through LASSO regression analyses, we identified 6 genes that exhibited significant associations with prognosis, including RPS6KA6, ABI3, PTTG1, E2F1, CBX7, and SPOP (Figure 4A, 4B). Table 1 shows the coefficients of each gene a. Using the prognostic model comprising these six DE-SRGs, we calculated the risk score for each patient, which consisted of 1 potential risky gene and 5 potential protective genes. According to the results of coefficient of each gene, we constructed the prognostic model as follows: risk score= -(PTTG1*0.009676657) –(E2F1*0.025364333) –(CBX7*0.054322881) –(SPOP*0.175131225) +(RPS6KA6*0.09447039)- (ABI3*0.070493701). Patients were then divided into low-risk (n=153) and high-risk groups (n=153) based on the median risk score. We further investigated the relationship among the 6 genes. The results indicated that they were significantly relevant (Figure 4C). Moreover, the heatmap (Figure 4D) depicted the expression levels of the six genes in both low- and high-risk patients within the TCGA dataset. Notably, we observed substantial differences between the high- and low-risk groups in relation to various clinical factors, including tumor status, lymph node metastasis (LNM), grade, menopause status, stage, age, and living status. We deeply analyzed the expression of the six genes in different kinds of samples. We found they were significantly different expressed in cervical tissues (Figure 4E). These results indicated the six senescence-related genes were significantly different in high and low risk groups.

**Table 1:** Six senescence associated genes and corresponding coefficient value.

**Senescence associated gene**	**Coefficient**
PTTG1	-0.0096767
E2F1	-0.0253643
CBX7	-0.0543229
SPOP	-0.1751312
RPS6KA6	0.09447039
ABI3	-0.0704937
Risk score	Low: <0.490
	High: ≥0.490

### Accurate evaluation of the 6 SRGs risk model for CC patients

**Figure 5:**
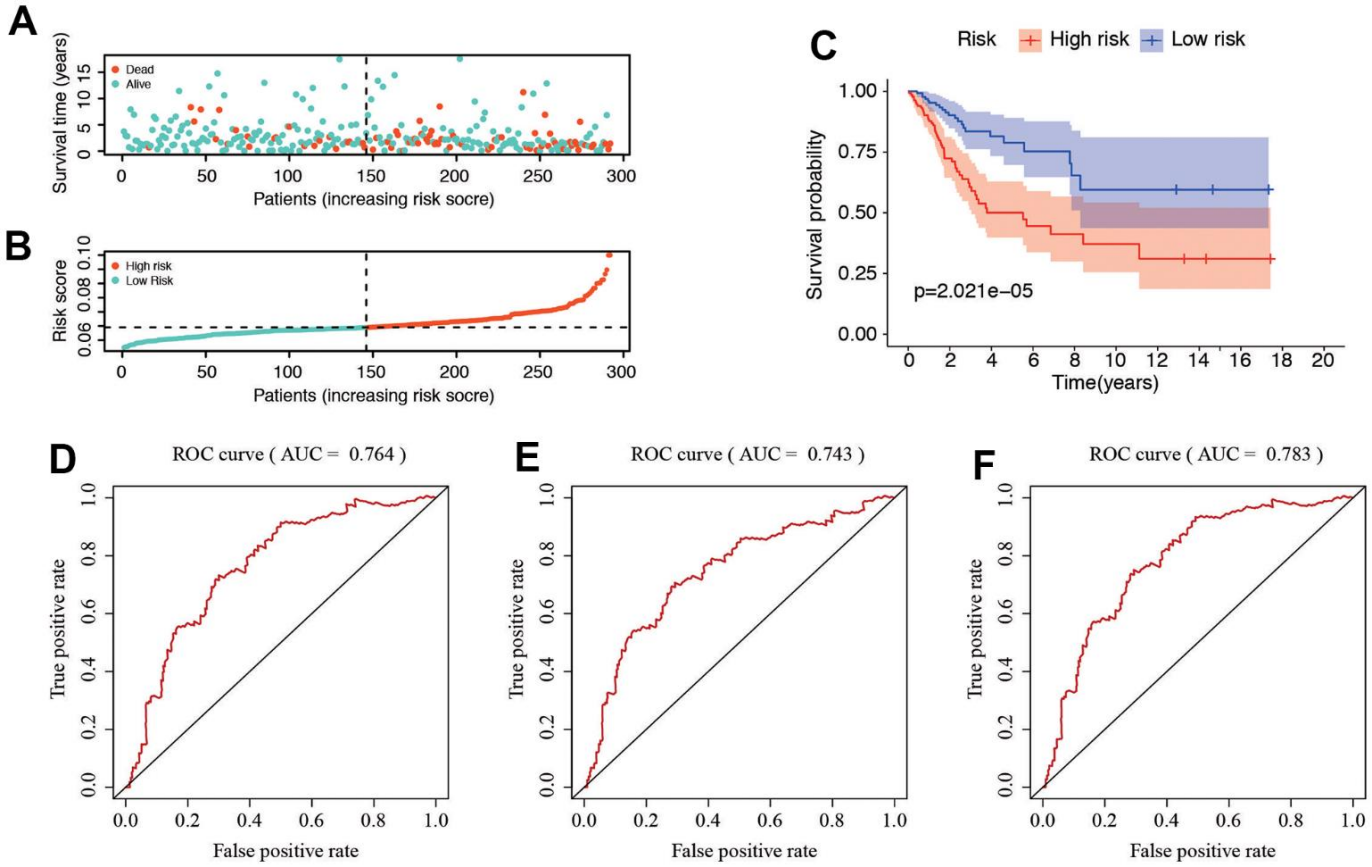
**Correlation between the risk score and clinicopathological features.** (**A**, **B**) Distribution of risk score and patient survival status of cervical cancer. (**C**) The Kaplan–Meier curve demonstrates that patients in the high-risk group have a poorer prognosis. (**D**–**F**) Time-dependent ROC curve of 1-, 3-, and 5-year analysis for survival prediction by the risk score.

The distribution of risk score and survival status of these 6 prognostic DE-SRGs are shown in Figure 5A, 5B. Patients in high-risk group were vulnerable to have a higher risk score and worse prognosis. The survival analysis using the Kaplan–Meier curve revealed a significantly worse prognosis for patients in the high-risk group (*P*=2.021e-05, Figure 5C). The time-dependent ROC analysis (Figure 5D–5F) displayed the predictive performance of the model, with AUC values of 0.764, 0.743, and 0.78 for predicting the 1-year, 3-year, and 5-year survival rates, respectively. These results suggested that the SRG-related risk model is accurate and high predictive for patients with CC.

### Function and enrichment analysis of the model

**Figure 6:**
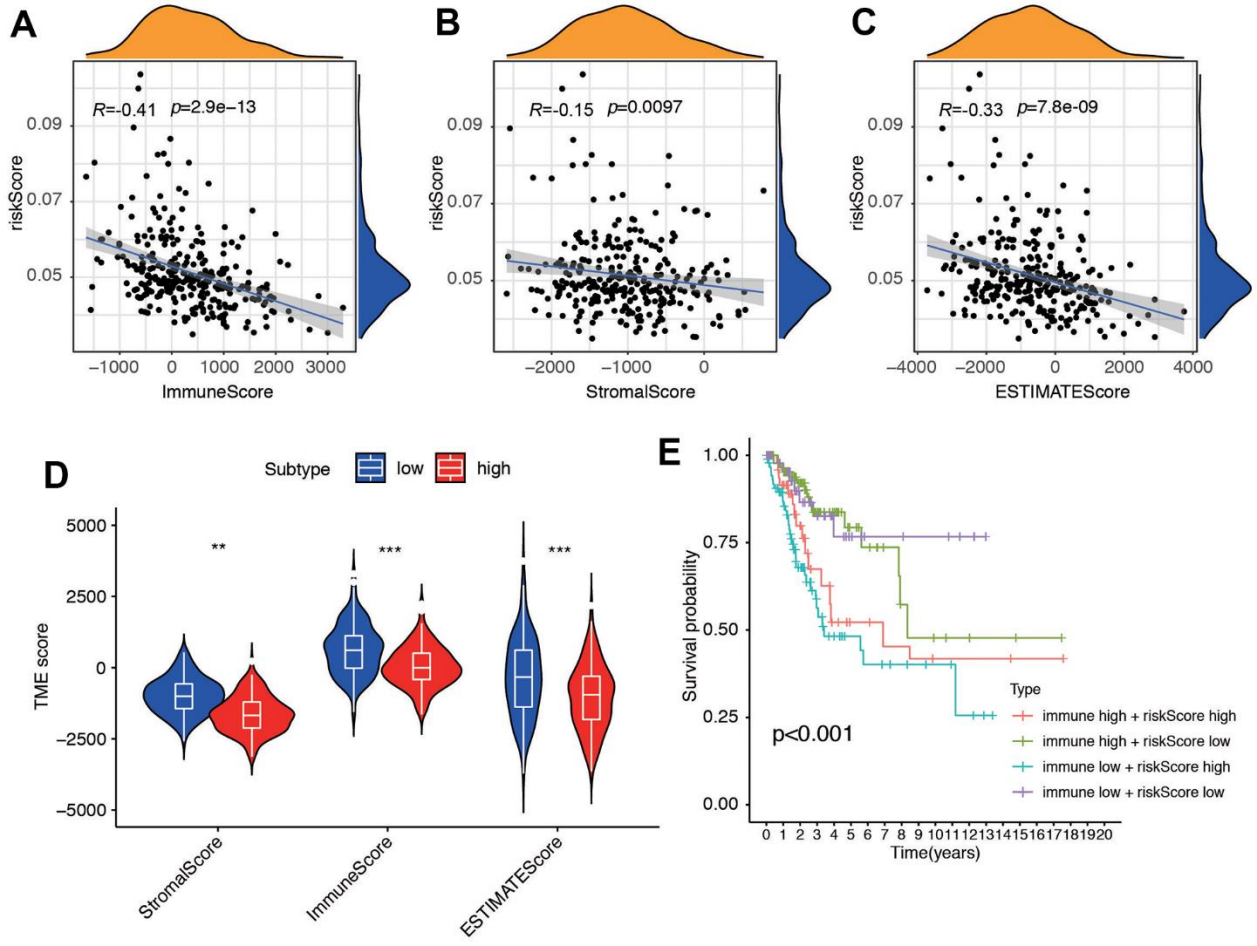
**The association between tumor microenvironment and risk score.** Correlation between (**A**) Immune Score, (**B**) Stromal Score and (**C**) ESTIMATE and risk score. (**D**) Different score between low- and high-risk groups. (**E**) Survival analysis for four groups stratified by combining the immune signature and the risk score characteristic in the TCGA-CC cohort.

We performed additional analysis to investigate the correlation between the risk score and ESTIMATE-related scores, encompassing immune score, stromal score, and ESTIMATE score. Our findings revealed a negative correlation between these scores and the risk score, with correlation coefficients of -0.41, -0.15, and -0.33, respectively (all *P* < 0.01, Figure 6A–6C), which pointed out that stromal and immune cell was lower in the high risk group. The results suggested that patients in the high-risk group had an unfavorable prognosis, which was associated with alterations in the tumor immune microenvironment of CC. We then compared the tumor microenvironment (TME) score between low and high risk groups (Figure 6D). Additionally, we categorized the patients into four subgroups based on their immune score and risk score, with high and low scores determined by the median value of the risk score. Conversely, patients with a low immune score and high risk score had the poorest prognosis (Figure 6E).

### Building and validation of the predictive nomogram

**Figure 7:**
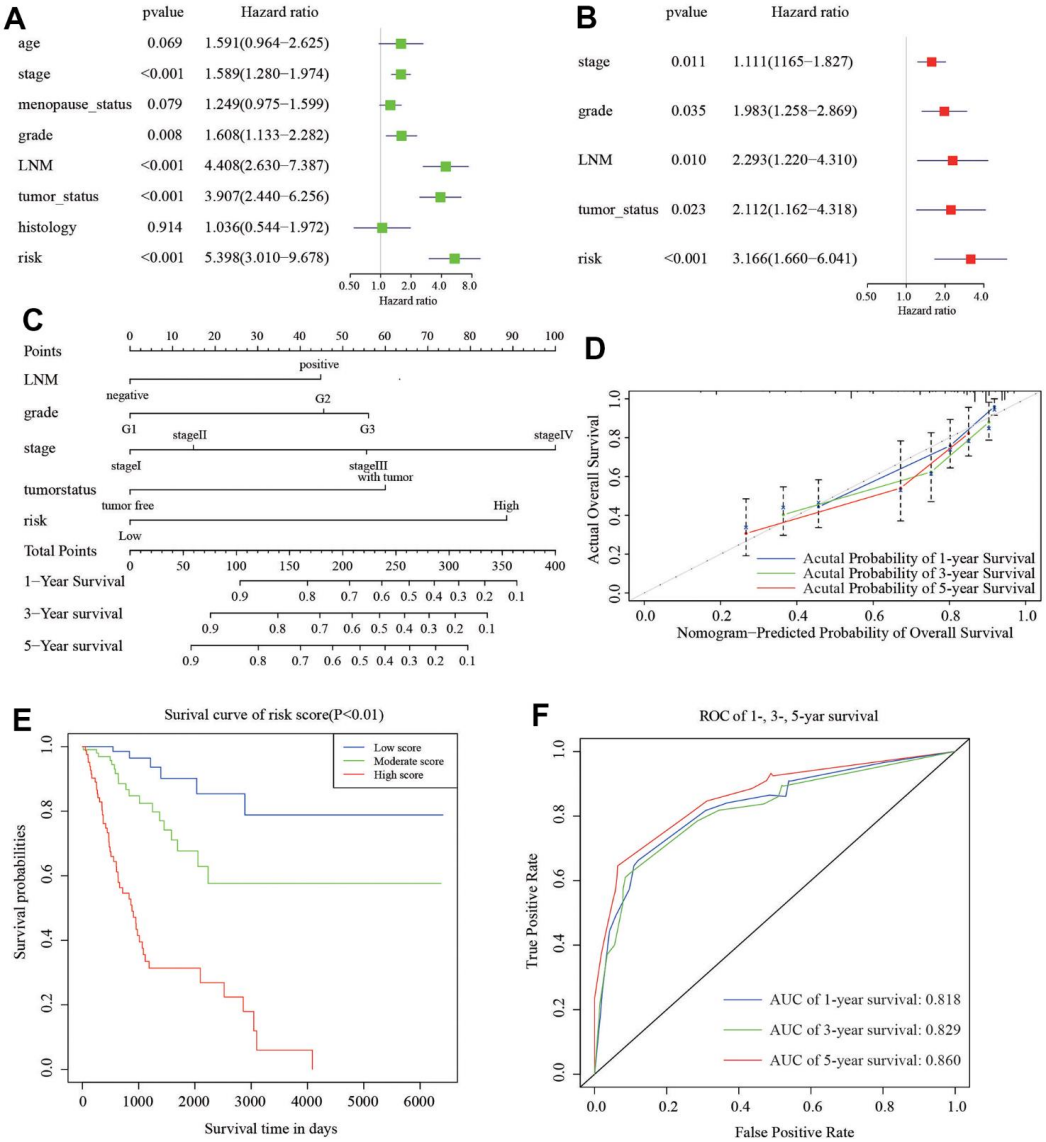
**Nomogram to predict the probability of patients with CC.** (**A**) Univariate and (**B**) multivariate regression analyses of the prognostic value of clinicopathological features. (**C**) The nomogram to predict 1-, 3-, or 5-year OS in the CC patients. (**D**) The calibration plots for predicting patient 1-, 3-, or 5-year OS. (**E**) The Kaplan–Meier curves represent the survival probability of low, moderate, and high score group patients based on the nomogram. (**F**) The ROC of 1-, 3-, 5-year survival curves by the nomogram.

To develop a clinical strategy for predicting the survival probability of CC patients, we created a nomogram using the TCGA cohort. This nomogram was used to assess the probability of different period of OS. We first performed the univariate and multivariate analyses to recognize independent risk factors for CC patients. The results of univariate analysis showed that stage (HR=1.589, 95%CI: 1.280-1.974, *P*<0.001), grade (HR=1.608, 95%CI: 1.133-2.282, *P*=0.008), LNM (HR=4.408, 95%CI: 2.630-7.387, *P*<0.001), tumor status (HR=3.907, 95%CI: 2.440-6.256, *P*<0.001), and 6-gene risk signature (HR=5.398, 95%CI: 3.010-9.678, *P*<0.001) are significantly associated with survival of CC (Figure 7A). Furthermore, we enrolled these factors into a multivariate Cox analysis. Finally, we found that stage (HR=1.111, 95%CI: 1.165-1.827, *P*=0.011), grade (HR=1.983, 95%CI: 1.258-2.869, *P*=0.035), LNM (HR=2.293, 95%CI: 1.220-4.310, *P*=0.010), tumor status (HR=2.112, 95%CI: 1.162-4.318, *P*=0.023), and 6-gene risk signature (HR=3.166, 95%CI: 1.660-6.041, *P*<0.001) were independent risk factors for CC patients (Figure 7B). These five independent prognostic factors were used to construct the nomogram (Figure 7C). The diagonal line at 45° represented the ideal prediction. The calibration plots demonstrated that the nomogram exhibited excellent performance in predicting the 1-year, 3-year, and 5-year survival outcomes (Figure 7D). The specific scores for each factor were provided in Table 2. The C-index of the model was 0.78 (95% CI: 0.69-0.86). Moreover, we divided the cohort evenly into three subgroups (low-score, moderate-score, and high-score groups) based on their risk scores from the nomogram. Subsequently, we evaluated the differences in Kaplan-Meier survival among these three groups, including the low-, moderate-, and high-risk groups. The survival curve of the high-score group demonstrated worse overall survival (OS) compared to the moderate and low-score groups (Figure 7E). ROC curve analysis in Figure 7F exhibited that the risk score AUC value of 1-, 3-, 5-year survival was 0.818, 0.829, and 0.860. These findings suggested that the nomogram had a high accuracy in predicting OS.

**Table 2:** Corresponding risk score for each variable and total score.

**Variables**	**Category**	**Score**
Age	<60	0
	≥60	35
LNM	Negative	0
	Positive	95
Grade	Negative	0
	Positive	40
Risk signature	Low	0
	High	100
Total score	Low risk	0-40
	Moderate risk	75-135
	High risk	≥140

### Biological features of significant genes found in this panel

**Figure 8:**
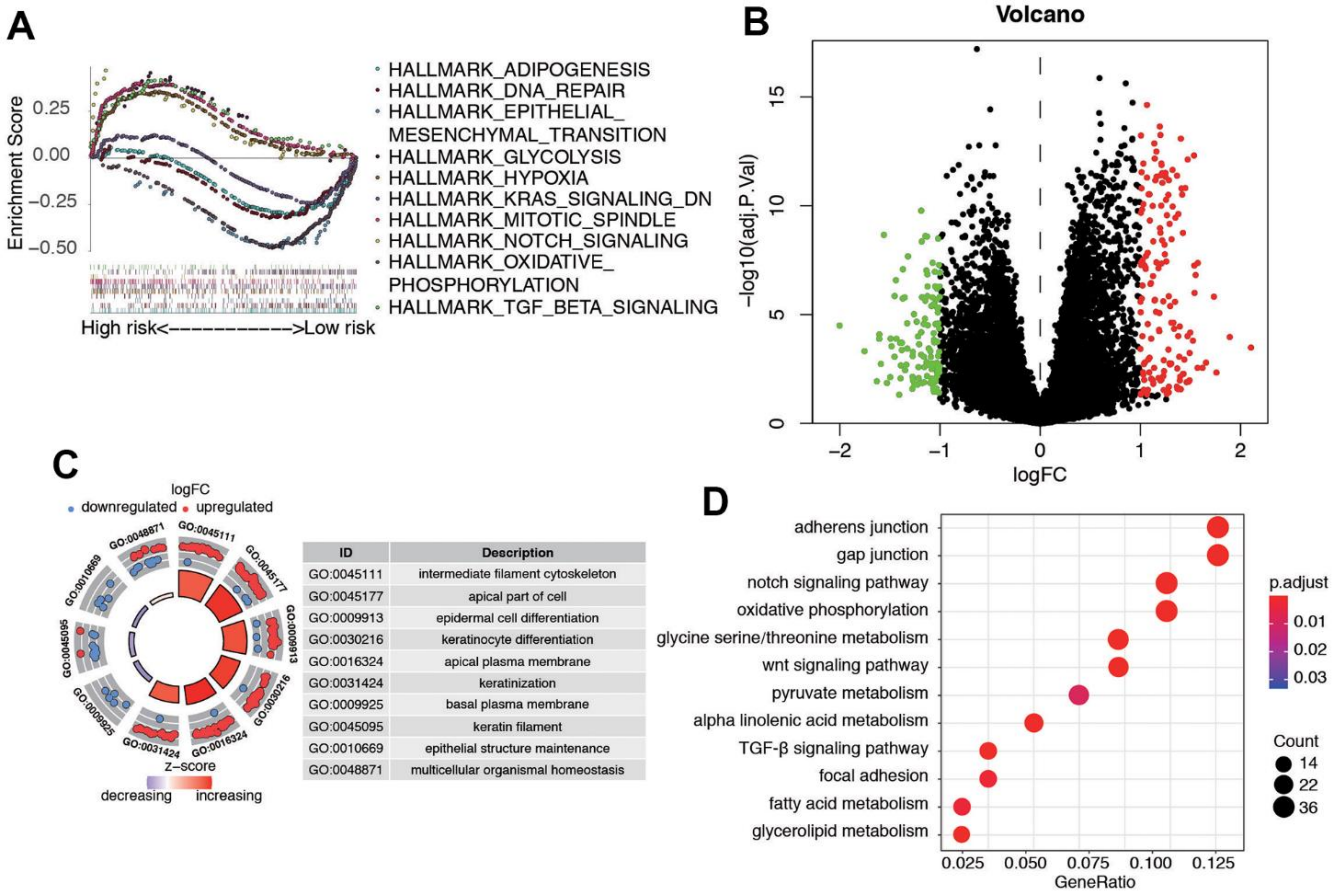
**Biological features and significant genes enrichment analysis in risk model.** (**A**) The GSEA enrichment analysis of senescence-related risk signature. (**B**) Heatmap of DEGs in different risk model. (**C**) The GO analysis of risk model-related DEGs. (**D**) The KEGG pathway analysis of risk model-related DEGs.

Furthermore, we utilized Gene Set Enrichment Analysis (GSEA) software to identify the enriched pathways or functions in the high-risk and low-risk patient groups. The top ten terms enriched in these groups are depicted in Figure 8A. The results showed that key important pathways, such as the adipogenesis, DNA repair, EMT, KRAS signaling, and Notch signaling were significantly activated in the risk group. Then, we performed a differential expression analysis between low-risk group and high-risk group using Wilcoxon test with a log_2_(Fold Change) > 1 and *P* < 0.05. We found 271 DEGs between the two groups, including 154 up-regulated genes and 117 down-regulated genes (Figure 8B). Supplementary Table 3 contains the DEGs list along with their corresponding log_2_FC and FDR adjusted *P*-values. Subsequently, we conducted GO and KEGG pathway analysis for these DEGs, and Figure 8C, 8D displays the top 10 enriched terms in the GO and KEGG pathways, respectively. The KEGG analysis indicated that the genes were mainly involved in gap junction, notch signaling pathway, oxidative phosphorylation, glycine serine/threonine metabolism, and WNT signaling pathway.

### External validation of the 6 genes

**Figure 9:**
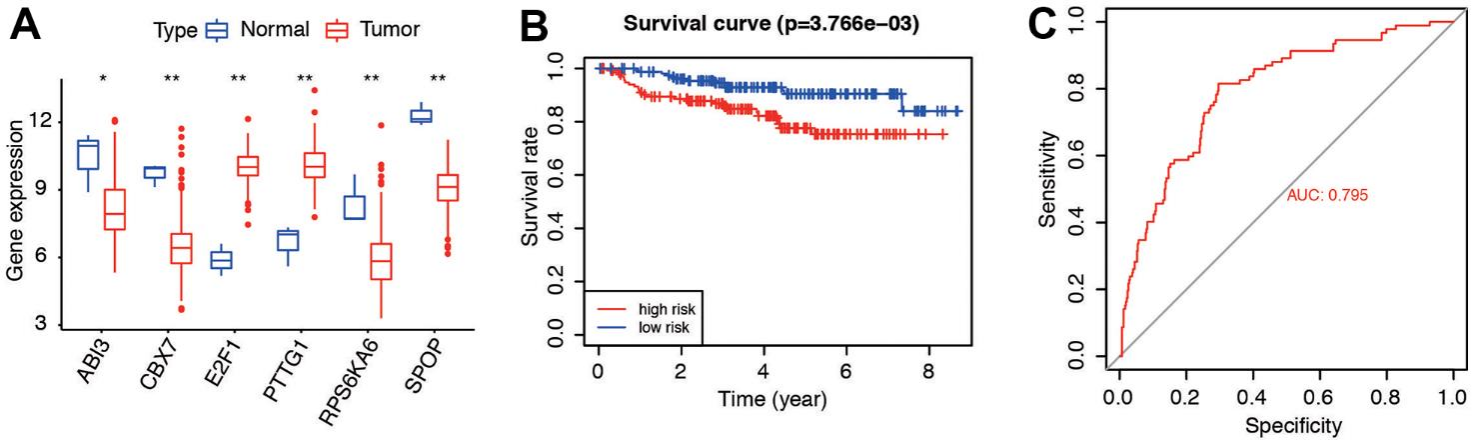
**External validation of the six genes and related risk model.** (**A**) Gene expression of the six senescence-related genes in GSE44001 dataset. (**B**) Survival curve of patients in low- and high-risk groups. (**C**) Predictive accuracy of the risk model in GSE44001.

GSE44001 and GSE52903 were acquired to validate. We compared the content of the six genes (Figure 9A), survival curve of risk model (Figure 9B, *P*=3.766e-03), and predictive accuracy by AUC (Figure 9C, AUC=0.795) in the dataset. Results in GSE52903 indicated the similar expression and prognosis (Supplementary Figure 1). Consistent with the results in the entire TCGA cohort, these results suggested that six genes were highly predictive for evaluating the prognosis of CC patients.

## DISCUSSION

The FIGO staging system is currently the widely accepted method for staging cervical cancer [13]. However, staging based on imaging and subjective judgment of doctors’ physical examination was not reliable and inaccurate sometimes if the patients were accompanied by complications. With the rapid development of oncogenesis and the discovery of biological factors that predict cancer outcome, some studies used biological markers to predict the prognosis of individual patient in cervical cancer.

In previous studies, risk model predicting survival of cervical cancer had been built based on some functional gene sets. Chen et al. developed a prognostic model based on a three-gene signature (SLAMF1, CD27, SELL) that is associated with the tumor microenvironment. The model exhibited a predictive accuracy with AUC values ranging from 0.71-0.78 [14]. In another study, an autophagy-related gene prognostic risk model was applied to provide a reference for CC patients to make precise treatment strategy, and the AUCs for prognostic accuracy reached 0.772 for the training set and 0.889 for the verification set. Other gene sets for establishing prognostic model in CC included immune-related genes [15], DNA methylation [16], and lymph node metastasis [17].

Senescence plays an important role in the development of different cancer types, which could lead to different outcomes for patients in the tumor microenvironment. SRGs act as prognostic markers for some cancers. Senescence is involved in the onset and development of various tumors, such as ovarian cancer, pancreatic cancer, renal cancer, and hepatocellular cancer [18–21]. However, the reported molecular mechanism of senescence is still limited. In this study, we aimed to identify the change in SRG status in CC patients and investigated the relationship between these SRGs and clinicopathological characteristics to see if they could help predict the clinical prognosis of patients. Our data provided useful information to better understand the biological changes in CC patients.

In this study, we extensively examined the role of SRGs in the advancement of cervical cancer by analyzing the mRNA data from patients with CC in the TCGA database. We obtained 74 dysregulated SRGs between normal and cancer tissues, among which six genes (RPS6KA6, ABI3, PTTG1, E2F1, CBX7, and SPOP) were associated with the OS of CC patients. The six SRGs showed a great accuracy in predicting the survival for CC patients, especially combining the SRG-related risk model with clinicopathological characteristics.

These six genes had been reported in some studies. One study analyzed the epigenomic landscape and identify RPS6KA6 as potential biomarker for CC, which is significantly correlated with the overall survival of CC [22]. Another study constructed a six-gene prognostic model, including PTTG1, that could become the new promising target for the cervical cancer treatment [23]. The function of PTTG1 might be modulated by the long non-coding RNA PTTG3P, and PTTG3P is upstream of PTTG1 [24]. It is reported that E2F Transcription Factor 1 activates FKBP Prolyl isomerase 4 to promote angiogenesis in cervical cell carcinoma via the PI3K/AKT signaling pathway [25]. CBX7 might also function as a tumor suppressor gene in cervical cancer, offering valuable insights for the diagnosis and potential targeted treatments of the disease [26]. SPOP promotes cervical cancer progression by inducing the movement of PD-1 away from PD-L1 in spatial localization [27]. However, ABI3 had not been reported in CC. ABI3 expression is frequently reduced or lost in most colon cancer cell lines, and interestingly, there exists a positive correlation between ABI3 and ABI3BP expression in these carcinomas [28]. The roles of the six genes in cervical cancer remain uncertain, and further research is warranted to gain a better understanding of their functions.

GSEA results found that high risk group was enriched in “adipogenesis”, “DNA repair”, “epithelial mesenchymal transition”, “glycolysis”, and “hypoxia”. Metabolic function contributed to progression of cervical cancer cells and modulated its metastasis [29]. FAD104 suppressed TGF-beta-mediated EMT in cervical cancer by regulating adipogenesis [30]. Hypoxia and glycolysis were often correlated with each other. Tian et al. showed that hypoxia-induced CNPY2 promoted glycolysis in cervical cancer cells by activating the AKT pathway [31]. These studies corresponded to the function of high-risk group in GSEA in our research.

GO and KEGG pathways were performed for DEGs. The results showed genes were mainly enriched in the function of cytoskeleton-related regulation. Reorganization of cytoskeleton promoted cell proliferation and metastasis in cervical via different signaling pathway [32, 33]. Rho guanosine triphosphatases (GTPases) signaling pathway was one of the most essential pathways that regulated actin cytoskeleton dynamics and mechanical activity central for cervical cancer cells. Rho GTPases in cervical cancer highlighted relevant signaling pathways and pathomechanisms, and shed light on their involvement in tumor progression, metastatic spread, and radio/chemo resistance [34].

Six SRGs were suitable to establish the prognostic gene signature models, which can be used to calculate the risk level for each patient. The statistics between high- and low-risk groups in Kaplan-Meier survival curves were significantly different. Data obtained from evaluation models demonstrated that the SRGs’ signature model is an effective method of evaluating the degree of patient risk. The AUC reached approximately 0.860 from the nomogram. Considering that the efficiency of a single biomarker was limited, we developed a multiple-gene signature that combined the risk score model and clinicopathological characteristics to improve the OS prognostic value in CC patients. Additionally, our externally validated stable gene expression in different types of samples indicated that the predictive capability of this nomogram in CC prognosis was reliable.

However, there are several limitations in this study. First, although the application of this risk model was verified by external validation, our data only conduct the expressional validation, and the model in this study should be validated by an external database. Second, the biological functions of senescence-related genes based on OS models need to be examined by a series of cellular function assay.

## CONCLUSIONS

In summary, our investigation has identified six SRGs (RPS6KA6, ABI3, PTTG1, E2F1, CBX7, and SPOP) that are linked to the development of CC. We have developed a 6-gene risk signature that can be utilized to predict the prognosis of cancer. Furthermore, this model has the potential to uncover novel therapeutic targets for advanced CC and facilitate personalized immunotherapy for patients. However, further in-depth research is necessary to fully comprehend the biological functions of these genes. The six genes hold promise as potential prognostic biomarkers and targets for therapy in cervical cancer.

## Supplementary Material

Supplementary Figure 1

Supplementary Table 1

Supplementary Table 2

Supplementary Table 3
