# Supplementary material for: A cellular senescence-related genes model allows for prognosis and treatment stratification of cervical cancer: a bioinformatics analysis and external verification
Source: Aging (Albany NY). 2023 Sep 27;15(18):9408–25. doi: 10.18632/aging.204981 (PMC10564413; doi:10.18632/aging.204981)
Supplement: Supplementary Figure 1 [file aging-15-204981-s001.pdf]

SUPPLEMENTARY FIGURE

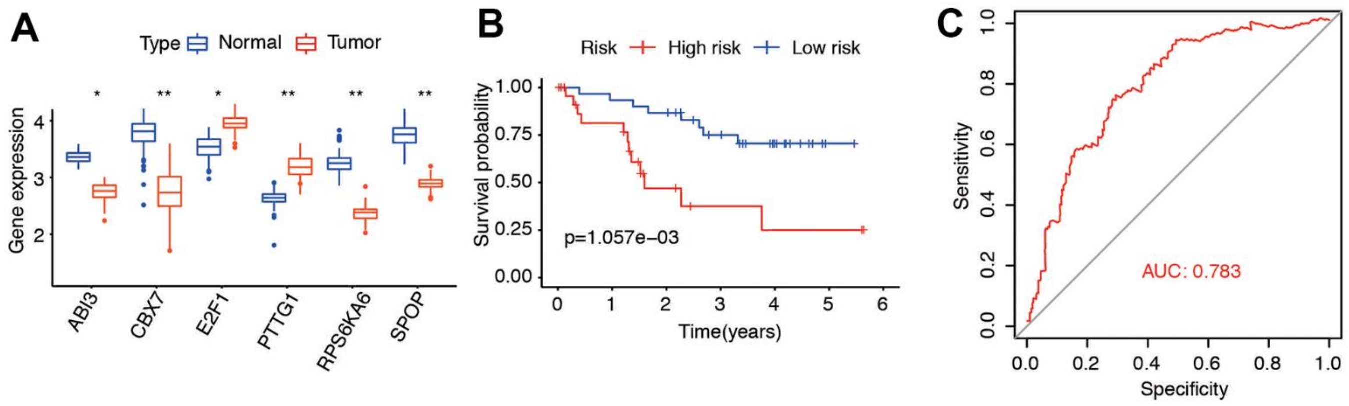

Supplementary Figure 1. External validation of the six genes and related risk model with GSE52903. (**A**) Gene expression of the six senescence-related genes in GSE52903dataset. (**B**) Survival curve of patients in low- and high-risk groups. (**C**) Predictive accuracy of the risk model in GSE52903.
