# Supplementary material for: A cellular senescence-related genes model allows for prognosis and treatment stratification of cervical cancer: a bioinformatics analysis and external verification
Source: Aging (Albany NY). 2023 Sep 27;15(18):9408–25. doi: 10.18632/aging.204981 (PMC10564413; doi:10.18632/aging.204981)
Supplement: Supplementary Table 2 [file aging-15-204981-s003.pdf]

Supplementary Table 2. List of 74 DEGs.

Name	logFC	P.Value	adj.P.Val
FOXM1	-5.86170516428349	4.46045945152081E-44	1.21770543026518E-41
HJURP	-5.57621129112412	1.36888180685155E-35	1.86852366635237E-33
CENPA	-5.43827417122037	2.11465261547137E-32	1.92433388007895E-30
CDK1	-3.8850623220383	2.73898824191886E-29	1.86935947510962E-27
AURKA	-3.87245750864836	1.15326659973837E-22	6.29683563457148E-21
EZH2	-4.10050592444439	1.25523128403732E-21	5.71130234236979E-20
PTTG1	-4.37795030662846	5.3847114167519E-19	2.10003745253324E-17
GNG11	4.62824060794036	1.86028991504963E-16	6.34823933510688E-15
HSPB2	5.11455890626433	2.29681119214258E-16	6.96699394949915E-15
CDKN2A	-6.58339612274595	1.44076146426149E-13	3.93327879743387E-12
E2F1	-3.44840608739512	2.33152080569296E-13	5.78641072685616E-12
SFN	-7.26192714058659	1.00693330519938E-12	2.29077326932859E-11
TACC3	-2.72369319320073	2.8906163783639E-12	6.0702943945642E-11
KIAA1524	-2.79022633808229	5.13139327196769E-12	1.0006216880337E-10
MAD2L1	-2.63639557430824	8.33078337521783E-12	1.51620257428964E-10
WT1	7.40700698583233	1.45728789277259E-11	2.48649746704323E-10
BRCA1	-2.33203746517941	4.81687921028178E-11	7.73534132004074E-10
STAT5B	2.1766933082377	1.09555012449645E-10	1.66158435548629E-09
TGFB1I1	4.22883977443808	1.245755300865E-10	1.7899536691376E-09
CBX7	3.28440230493961	7.44550770102602E-10	1.01631180119005E-08
DHCR24	-3.24155329581328	1.57391371415992E-09	2.0460878284079E-08
MYLK	5.19926212987162	1.98405945269496E-09	2.46203741175329E-08
RUNX1	-2.36344059617358	1.02150463418587E-08	1.21248158753366E-07
ITPKB	2.67776838806486	1.12673355433192E-08	1.28165941805255E-07
FBXO31	1.52702626261167	1.69234503978028E-08	1.84804078344007E-07
CHEK1	-2.00254625277945	2.00868556684407E-07	2.10911984518627E-06
SPOP	1.22634315842419	8.67311274752387E-07	0.00000876948066694 08
EPHA3	4.3815270582792	0.0000010777431126169	0.0000105079953480148
TBX2	3.16732073733568	0.0000083334933819594	0.0000784497825267212
GAPDH	-1.31693143515505	9.77368624185925E-06	0.0000889405448009192
KL	3.2846750445521	0.0000104541520304156	0.0000895706258829007
EHF	-4.75901922185735	0.0000104991209826111	0.0000895706258829007
VENTX	2.70039215760127	0.0000158696174351155	0.000131285016963228
PTRF	2.98628346059878	0.0000171741815684357	0.000137898575534792
PSMD14	-1.12551189849044	0.0000196142465831077	0.00015299112334824
SOX5	3.35059110963594	0.0000253075378739793	0.000191915495544343
NOX4	2.72911150223548	0.00004305922693185	0.000317438459370071
DUSP3	1.1952799876478	0.0000441855730991308	0.000317438459370071
AR	3.88881360835097	0.000050100246269044	0.000350701723883308
TFDP1	-1.47186058183875	0.0000599779804329606	0.000409349716454956
SYK	-2.23211404692609	0.000247568418859178	0.00164844337435502
SREBF1	-1.88866165759232	0.000280520090583452	0.00182338058879244
HDAC4	1.6890127585392	0.000334829600957266	0.00212577862933334
CYR61	3.05183447805601	0.000455036622282331	0.00282329540643355
NEK1	1.38287842333243	0.000519761955963935	0.00315322253284787
IGFBP5	4.26217413429885	0.000607769991809902	0.0035130076951236
RPS6KA6	2.56306203111084	0.000612744761334167	0.0035130076951236
WNT2	3.34175216619456	0.000627804071890863	0.0035130076951236

MXD4	1.43291112045647	0.000704200073462239	0.00373757450310925
IGFBP6	3.67489026573696	0.000710577505923215	0.00373757450310925
FOXO3	1.14888506020765	0.00071191895297319	0.00373757450310925
AGT	3.98960623841807	0.000749358252368261	0.00385990194144406
CAV1	2.92919121092568	0.000869064162041928	0.0043936021525453
TERT	-3.15618419221303	0.000916102544880707	0.00454719990458969
SNAI1	2.42850949230052	0.000949254171547842	0.00462761408629573
SMARCA4	-1.12793473621185	0.00110024695727781	0.00526960384801478
CPEB1	3.48279491141759	0.0013509751140298	0.00635890010569198
MMP9	-3.31361473067005	0.00140794298854971	0.00651471925210288
CDKN1C	2.67727438524094	0.00170263515401685	0.00774698995077668
ID4	2.99751486853733	0.00178242702394679	0.00797709143504054
SIRT1	1.15411229460634	0.00235678744742038	0.0100531714554025
NUAK1	2.29624836223771	0.00247443732828889	0.0102483952462883
CDKN2B	-2.39961157162258	0.00247763401558618	0.0102483952462883
TXN	-1.45142678530736	0.00284687554858605	0.0115999555934924
AXL	2.19681830967857	0.00378093154216189	0.0151793281030911
TXNIP	2.06396712361334	0.00514441922348382	0.019616025688164
ZFP36	1.82357682242161	0.00517345732435095	0.019616025688164
ITGB4	-1.85167125775667	0.00884455099488515	0.0326292219135628
ABI3	1.74272266086465	0.00910740188548874	0.0331043307859831
SOX2	-4.92563549009624	0.00921585765470592	0.0331043307859831
PLA2R1	2.12080039907351	0.0110449468271901	0.0385582804524716
PRKCD	-1.04542542951809	0.0113633479146101	0.0387774247586071
TP63	-5.2600041356328	0.0132777117272212	0.0442050646528218
IGFBP1	1.9258450115148	0.0141110551206854	0.0464134704571942
